# Amplification of HER2 is a marker for global genomic instability

**DOI:** 10.1186/1471-2407-8-297

**Published:** 2008-10-14

**Authors:** Rachel E Ellsworth, Darrell L Ellsworth, Heather L Patney, Brenda Deyarmin, Brad Love, Jeffrey A Hooke, Craig D Shriver

**Affiliations:** 1 Clinical Breast Care Project, Henry M. Jackson Foundation for the Advancement of Military Medicine, Rockville, MD, USA; 2 Clinical Breast Care Project, Windber Research Institute, Windber, PA, USA; 3 Invitrogen, Carlsbad, CA, USA; 4 Clinical Breast Care Project, Walter Reed Army Medical Center, Washington, DC, USA

## Abstract

**Background:**

Genomic alterations of the proto-oncogene c-erbB-2 (HER-2/neu) are associated with aggressive behavior and poor prognosis in patients with breast cancer. The variable clinical outcomes seen in patients with similar HER2 status, given similar treatments, suggests that the effects of amplification of HER2 can be influenced by other genetic changes. To assess the broader genomic implications of structural changes at the HER2 locus, we investigated relationships between genomic instability and HER2 status in patients with invasive breast cancer.

**Methods:**

HER2 status was determined using the PathVysion^® ^assay. DNA was extracted after laser microdissection from the 181 paraffin-embedded HER2 amplified (n = 39) or HER2 negative (n = 142) tumor specimens with sufficient tumor available to perform molecular analysis. Allelic imbalance (AI) was assessed using a panel of microsatellite markers representing 26 chromosomal regions commonly altered in breast cancer. Student t-tests and partial correlations were used to investigate relationships between genomic instability and HER2 status.

**Results:**

The frequency of AI was significantly higher (*P *< 0.005) in HER2 amplified (27%) compared to HER2 negative tumors (19%). Samples with HER2 amplification showed significantly higher levels of AI (*P *< 0.05) at chromosomes 11q23, 16q22-q24 and 18q21. Partial correlations including ER status and tumor grade supported associations between HER2 status and alterations at 11q13.1, 16q22-q24 and 18q21.

**Conclusion:**

The poor prognosis associated with HER2 amplification may be attributed to global genomic instability as cells with high frequencies of chromosomal alterations have been associated with increased cellular proliferation and aggressive behavior. In addition, high levels of DNA damage may render tumor cells refractory to treatment. In addition, specific alterations at chromosomes 11q13, 16q22-q24, and 18q21, all of which have been associated with aggressive tumor behavior, may serve as genetic modifiers to HER2 amplification. These data not only improve our understanding of HER in breast pathogenesis but may allow more accurate risk profiles and better treatment options to be developed.

## Introduction

The HER2 (c-*erb*-B2, HER-2/*neu*) gene, located on chromosome 17q12, is a member of the epidermal growth factor receptor family with tyrosine kinase activity [1]. Amplification of the HER2 gene and/or over-expression of the corresponding protein have been detected in 15–25% of human breast cancers and is associated with poor prognosis [2,3]. Under current standards of clinical care, patients with HER2 amplified (HER2+) tumors receive trastuzumab in combination with standard chemotherapy [4], however, despite treatment with trastuzumab, many HER2+ patients develop distant and progressive metastatic disease [5,6].

To develop more effective treatments for patients with HER2+ breast tumors, efforts have focused on the identification of genes that modify clinical response to trastuzumab including cyclin-dependent kinase inhibitor 1B (p27), phosphatase and tensin homolog (PTEN), insulin-like growth factor 1 receptor (IGF1R) and topoisomerase II α (TOP2A) [7-11]. In addition, efforts to characterize molecular changes associated with HER2 amplification revealed a cluster of genes from the 17q12-q21 region with similar patterns of amplification, and concordant changes in gene expression [12-14]. Furthermore, multiple gene expression analyses have defined molecular signatures for breast tumors with varying pathological characteristics [13,15,16], improving the ability to accurately characterize tumor sub-types.

Despite advances in molecular characterization of HER2+ breast tumors, mechanisms by which HER2 amplification contributes to breast cancer pathogenesis remain unknown. In this study, we examined levels and patterns of allelic imbalance (AI) in primary breast tumors with and without HER2 gene amplification to 1) examine associations between amplification of the HER2 gene and global genomic instability and 2) identify chromosomal changes commonly observed in HER2 amplified tumors.

## Methods

Paraffin-embedded primary breast tumors were obtained from the Windber Medical Center Pathology Department or the Clinical Breast Care Project (CBCP) Pathology Laboratory. Samples from patients with a previous history of breast cancer or who had received neoadjuvant therapy were excluded from this study. Samples from the Windber Medical Center were archival in nature, having been diagnosed between 1991 and 2003; clinical information was provided for these de-identified samples by the Memorial Medical Center Cancer Registry. Tissue and blood samples from CBCP patients were collected between 2001 and 2006 with approval from the Walter Reed Army Medical Center Human Use Committee and Institutional Review Board. All subjects enrolled in the CBCP voluntarily agreed to participate and gave written informed consent. Clinical information was collected for all CBCP samples using questionnaires designed by and administered under the auspices of the CBCP.

To ensure consistency, diagnosis of all tumor samples were made by one pathologist from hemotoxylin and eosin (H&E) stained slides; staging was performed using guidelines defined by the AJCC *Cancer Staging Manual *sixth edition [17,18]. HER2 status was assayed using the PathVysion^® ^HER-2 DNA Probe kit (Abbott Laboratories, Downers Grove, IL). Amplification was defined as a HER2:CEP 17 signal ratio of ≥ 2.2 [3]. Patients with either equivocal HER2 status (1.8 – 2.2) or aneusomy were not evaluated in this study. Clinicopathological information for all samples is summarized in Table 1.

**Table 1:** Clinical and pathological characteristics of 181 invasive breast tumors at the time of diagnosis

	**HER2 amplified (n = 39)**	**HER2 negative (n = 142)**	**P-value**^a^
***Menopausal Status***			
Pre (<50 years)	31%	31%	NS
Menopausal (≥ 50 years)	69%	69%	
***Tumor Type***			
Infiltrating ductal	84%	73%	NS
Infiltrating lobular	8%	16%	
Mixed ductal and lobular	5%	5%	
Other	3%	6%	
***Tumor Grade***			
Well (Grade 1)	11%	41%	P < 0.0001^b^
Moderate (Grade 2)	26%	36%	
Poor (Grade 3)	63%	23%	
***Hormone Receptor Status***			P < 0.05^c^
ER+/PR+	53%	63%	
ER+/PR-	11%	17%	
ER-/PR+	0%	3%	
ER-/PR-	36%	17%	
***Lymph Node Status***			NS
Negative	47%	53%	
Positive	53%	47%	
***TNM Stage***			NS
Stage I	36%	47%	
Stage II	40%	35%	
Stage III	21%	16%	
Stage IV	3%	2%	
***HER2 – IHC***			P < 0.0005^d^
0+	0%	17%	
1+	6%	33%	
2+	17%	49%	
3+	77%	1%	

^a^P-values calculated for HER2 positive versus negative tumors.

^b^Comparison between well- and poorly-differentiated tumors.

^c^Comparison between hormone receptor negative (ER-/PR-) and hormone receptor positive tumors.

^d^Comparison between 0+/1+ and 2+/3+.

**Figure 1:**
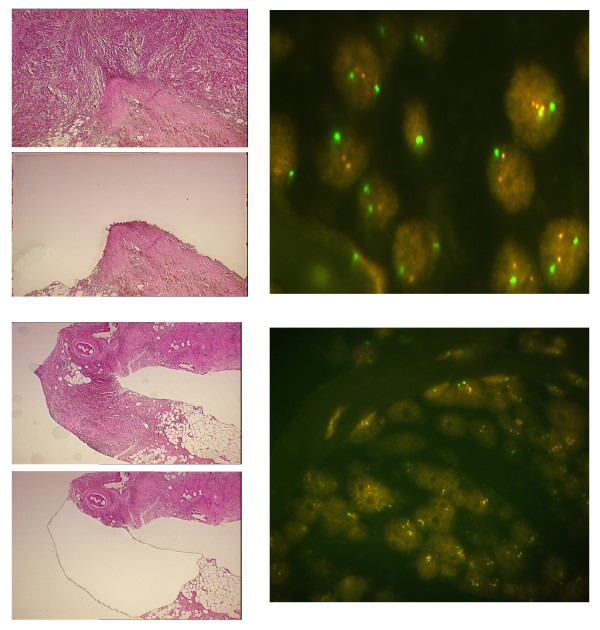
**Images of tumors before and after laser-assisted microdissection with corresponding FISH data.** The tumor specimen on the top was taken from a pre-menopausal woman with stage IIb IDCA, without amplification of the HER2 gene. The tumor specimen on the bottom was taken from a pre-menopausal woman stage IIIB IDCA and HER2 amplification of 3.3. Green signals = CEP17 probe, orange = HER2.

DNA was obtained from pure populations of primary breast tumor cells following laser-assisted microdissection on an AS *LMD *laser microdissection system (Leica Microsystems, Wetzlar, Germany) [19] (Figure 1). All microdissected sections were examined by the CBCP pathologist, who identified and marked regions of tumor before dissection. To avoid PCR artifacts, ≥ 5,000 cells were captured from each of six consecutive breast tumor sections, with the sixth section reserved for all confirmatory reruns. Referent DNA samples for the archival samples were extracted from disease-free skin or negative lymph node tissue from each patient using the QIAamp DNA Mini Kit (Qiagen, Valencia, CA). Referent DNA for the CBCP samples was obtained from blood clots using Clotspin and Puregene DNA purification kits (Gentra, Minneapolis, MN).

**Figure 2:**
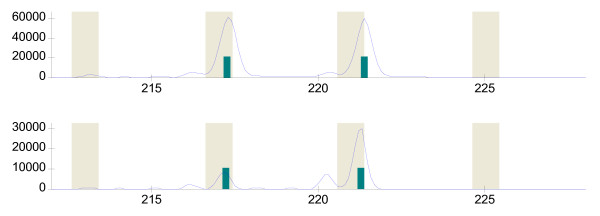
**Detection of allelic imbalance in a HER2+ breast tumor with fluorescence-based genotyping.** Alleles for marker D17S250 on chromosome 17q12 were detected as fluorescent peaks in reference DNA (top panel) and microdissected breast tumor DNA (bottom panel). A normalized peak height ratio of 0.28 was calculated for the tumor sample using the following peak heights in relative fluorescence units (rfu): tumor DNA – 8,791 rfu and 30,585 rfu, referent DNA – 61,195 rfu and 59,934 rfu.

Microsatellite markers were amplified as previously described [20], purified using Sephadex G-50 resin and genotyped on a MegaBACE-1000 capillary electrophoresis apparatus (Amersham Biosciences, Piscataway, NJ) following standard protocols. Genotypes were determined using Genetic Profiler version 2.0 software. AI was detected as previously described [21] using a cutoff value of 0.35 (Figure 2), which provides >80% reproducibility when AI events are confirmed on a second aliquot of DNA [22].

To increase the ability to detect AI, two microsatellite markers from each chromosomal region were assayed. Marker information was then pooled and AI at each chromosomal region was defined as follows: 1) when at least one marker for a given region showed an allelic ratio ≤ 0.35, the region was considered to show AI; 2) when neither marker had an allelic ratio ≤ 0.35 and at least one marker was informative, the region was considered normal; and 3) when both markers were homozygous, the region was considered uninformative.

Comparison of the clinicopathological factors and levels and patterns of AI by HER2 status were performed using Mann-Whitney and Fisher's exact tests. Potential confounding factors were investigating by computing the significance of partial correlations between HER2 and AI while holding grade and ER constant as mitigating factors. Correlations were calculated non-parametically from the 2 by 2 tables for ordinal scores (using Phi coefficient of association) and then a direct application of the partial correlation calculation was conducted. P-values were estimated from the non-parametric partial correlation using parametric assumptions to attempt to indicate if true correlation was observed. A significance value of *P *< 0.05 was used for all analyses.

## Results

### Clinicopathologic features

In total, 181 samples were included in this study. All samples were collected from female patients; 22% (n = 39) were HER2+. While age at diagnosis, lymph node status, tumor histology and stage did not differ significantly between the two groups, poor differentiation and hormone receptor negative status were observed significantly more frequently in HER2+ compared to HER2- tumors. Ninety-four percent of HER2 amplified samples had IHC scores of 2+ or 3+.

Of the 39 HER2+ tumors, six were from patients diagnosed with invasive breast cancer prior to 1998, thus treatment with trastuzumab was not available for these patients. Of the remaining 33 HER2+ patients, ten were treated with trastuzumab (Table 2). Four HER2+ patients have died of disease and three have developed distant metastasis. The large number of specimens collected within the last 5 years precluded the analysis of outcome data in this study.

**Table 2:** Clinical characteristics of 39 HER2+ patients

Sample	Date Diagnosis	Stage Diagnosis	Trastuzumab	Status^a^
1	2003	IIA	No	NED
2	2003	IIB	No	DOD
3	2003	IIA	Yes	NED
4	2003	IIA	No	NED
5	2005	I	Yes	NED
6	2003	IIA	Yes	DOD
7	2004	IIA	No	NED
8	2004	I	No	NED
9	2004	IV	Yes	AWD
10	2004	IIIA	Yes	AWD
11	2004	IIIA	Yes	NED
12	2004	IIIC	Yes	AWD
13	2004	I	No	NED
14	2004	I	No	NED
15	2004	IIIC	Yes	NED
16	2004	IIIA	Yes	NED
17	2001	IIB	No	NED
18	2001	I	No	NED
19	2002	I	No	NED
20	2002	IIA	No	UNK
21	2002	IIIA	No	NED
22	2002	I	No	NED
23	2002	I	No	NED
24	2002	IIB	No	NED
25	2003	IIIC	No	NED
26	2003	IIA	No	NED
27	2005	I	No	NED
28	2004	IIA	No	NED
29	2005	IIIA	No	NED
30	2005	I	No	NED
31	1996	IIIB	No	DOC
32	2002	I	Unknown	NED
33	2003	I	Yes	NED
34	1992	IIA	No	NED
35	1993	IIIA	No	DOD
36	1994	I	No	NED
37	1995	I	No	DOC
38	1995	I	No	DOD
39	1996	IIB	No	NED

^a^AWD = alive with disease, DOC = dead other causes, DOD = dead of disease, NED = no evidence of disease, UNK = status unknown

### Genetic differences in HER2+ and HER2- tumors

Median AI levels were 25% (range 0–80%) and 13% (range 0–67%) in HER2+ and HER2- tumors, respectively. Levels of AI were significantly higher (*P*<0.005) in HER2 amplified (mean = 27%) compared to HER2 negative (mean = 19%). When stratified by chromosomal region, AI events were detected significantly more frequently in HER2+ tumors at 11q23, 16q22-q24 and 18q21 (Table 3). No region was altered at significantly higher levels in HER2- tumors.

**Table 3:** Frequency of AI by HER2 status at 26 chromosomal regions

Chromosomal Region	HER2+	HER2-	*P *HER2+ v HER2-
1p36.1-p36.2	0.25 (36)	0.12 (140)	0.0658
2q21.3-23.3	0.14 (36)	0.15 (130)	1.0000
3p14.1	0.19 (36)	0.18 (129)	0.8100
5q21.1-q21.3	0.23 (35)	0.16 (133)	0.3233
6q15	0.18 (38)	0.20 (133)	1.0000
6q22.1-q23.1	0.25 (37)	0.16 (134)	0.2286
6q25.2-q27	0.32 (37)	0.20 (136)	0.1219
7q31.1-q31.31	0.20 (35)	0.08 (133)	0.0523
8p22-p21.3	0.26 (35)	0.18 (130)	0.3476
8q24	0.26 (39)	0.16 (129)	0.1577
9p21	0.20 (35)	0.12 (129)	0.2607
10q23.31-q23.33	0.14 (37)	0.15 (132)	1.0000
11p15	0.26 (35)	0.18 (133)	0.3411
11q13.1	0.33 (39)	0.19 (137)	0.0790
11q23	0.50 (36)	0.23 (128)	**0.0033**
13q12.3	0.27 (37)	0.30 (130)	0.6641
13q14.2-q14.3	0.29 (39)	0.18 (133)	0.1790
14q32.11-q31	0.18 (38)	0.20 (131)	1.0000
16q11.2-q22.1	0.30 (37)	0.30 (134)	1.0000
16q22.3-q24.3	0.50 (36)	0.26 (129)	**0.0076**
17p13.3	0.43 (30)	0.26 (117)	0.0789
17p13.1	0.33 (39)	0.28 (137)	0.5564
17q12-q21	0.26 (38)	0.17 (133)	0.2367
18q21.1-q21.3	0.27 (37)	0.13 (128)	**0.0416**
22q12.3	0.27 (38)	0.15 (132)	0.4565
22q13.1	0.33 (39)	0.26 (134)	0.6639

Numbers in bold are statistically significant

### Confounding factor analysis of AI and HER2 with ER status and tumor grade

Because ER status and tumor grade have been associated with HER2 status and may confounding the relationship between HER2 amplification and AI, partial correlations were calculated at each chromosomal region between HER2+ and HER2- samples, holding ER status and grade constant. While the correlation between AI and HER2 status at chromosome 11q23 was influenced by grade, three chromosomal regions, 11q13.1, 16q22-q24 and 18q21 showed significant correlations with HER2 status that cannot be explained by either ER status or grade.

## Discussion

The development of trastuzumab has been cited as a successful example of pharmacogenomics where treatment choices are personalized to individual patients based on specific tumor characteristics. Not all patients with HER2+ tumors, however, will benefit from trastuzumab, and given that the cost of trastuzumab ranges from $20,000 – $80,000/year with potential side effects including fever and chills, gastrointestinal toxicity, myelosuppression, and cardiotoxicity with heart failure [23], more precise prediction of which HER2+ patients will derive benefit from trastuzumab and improved understanding of how amplification and/or overexpression of HER2 contribute to aggressive tumor biology are critical to improving patient treatment.

Breast cancer pathogenesis is associated with an accumulation of sequential genetic alterations. Early genetic changes that deregulate tumor suppressor and oncogenes may render these cells susceptible to additional genetic damage and lead to widespread instability in the tumor genome. Increasing levels of genetic changes have been associated with adverse characteristics such as poorly differentiated pathology, metastasis and decreased survival [24-26]. Isola et al. assessed global copy number changes using comparative genomic hybridization and found that tumor with HER2 amplification had significantly higher levels of aberrations compared to HER2- tumors, suggesting that these tumors were genetically more advanced [27]. In agreement with the results of Isola et al., we found that higher levels of chromosomal alterations were correlated with HER2 status, suggesting that HER2 amplification may serve as a surrogate marker for underlying genomic instability

In addition to the high levels of overall genomic instability associated with HER2 amplification, the positive association between chromosomal alterations at chromosomes 11q13.1, 16q22-q24 and 18q21 and HER2 amplification suggests that genes in these regions may contribute to pathogenesis of HER2+ tumors. Bertucci et al. identified a 36-gene expression profile of HER2+ breast tumors which included altered expression of genes chromosomes 11q and 16q, including the fatty acid desaturase 2 (FADS2) gene [GenBank: AF084559] on chromosome 11q12.2 and the M-cadherin (CDH15) gene [GenBank: D83542] on chromosome 16q24 [28]. Deletions of chromosome 18q have been associated with poor survival [29]. Loss of chromosome 18q has been associated with amplification of HER2 as well tumor progression and poor prognosis [27,29,30]. Thus, chromosomal alterations at these regions may contribute to the aggressive pathology and poor prognosis associated with HER2+ tumors.

**Figure 3:**
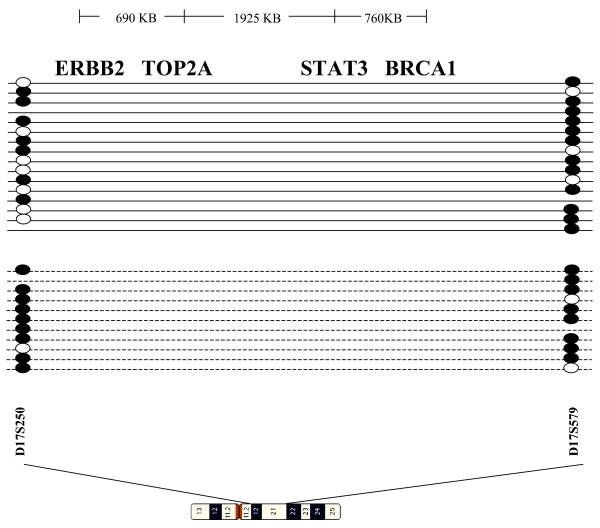
**Schematic diagram of the 17q12-q21 region.** Genes involved in breast cancer are listed across the top, with distance between genes noted. Patterns of AI are as follows: solid line = HER2- tumors, dotted line = HER2 amplified. Black circles represent an AI event, white circles, normal chromosomal content. Where there is no circle, the reference genotype was homozygous, and thus uninformative.

Because the HER2 gene is located within one of the 26 regions in our AI panel, patterns of AI at 17q12-q21 were examined. The two markers used to assess chromosomal content at 17q12-q21, D17S250 and D17S579, define a 5.7Mb region that includes not only the HER2 and TOP2A genes but also the signal transducer and activator of transcription 3 (STAT3) and breast cancer 1 (BRCA1) genes (Figure 3). In samples with AI at chromosome 17q12-q21, the majority of HER2- cases had AI at either D17S250 (24%) or D17S579 (59%), while in 50% of HER2+ tumors, AI was detected at both markers, suggesting that multiple genes from the 17q region may be altered in HER2+ tumors.

TOP2A alterations may contribute to pathogenesis in HER2+ breast carcinomas by altering sensitivity to anthracyclines [31]. Increased expression of STAT3, which may alter cellular proliferation, angiogenesis, and apoptosis, can be activated by HER2 [32,33], and has been associated with advanced disease and poor response to chemotherapy [34], thus genomic amplification of 17q12-q21 may increase STAT3 levels and contribute to aggressive, refractory breast disease. Although mutations in BRCA1 are uncommon in sporadic cancers, physical loss of BRCA1 has been detected in ~50% of sporadic tumors. BRCA1 deletions may be attributed to the genomic structure of BRCA1, which is characterized by unusually high numbers of *Alu *and non-*Alu *repetitive sequences [35]. During replication, mis-pairing between repetitive elements can lead to large deletions in the BRCA1 gene region [36]. How the unstable genomic structure of BRCA1 affects HER2 is unknown, however, because BRCA1 is a DNA repair enzyme, deletions of BRCA1 may impair cellular DNA repair, leading to an accumulation of DNA damage across the genome.

## Conclusion

In conclusion, breast tumors with copy number changes in the HER2 gene show higher levels of overall genomic instability. Alterations at chromosomes 11q13, 16q22-q24 and 18q21, all of which have been associated with aggressive tumor behavior, may serve as genetic modifiers to HER2 amplification. In addition, alterations within and across the 17q12-q21 region, including TOP2A, STAT3 and BRCA1 may modify the effects of HER2 amplification. Future studies to identify the gene alterations associated with HER2 amplification as well as examination of a larger group of HER2+ patients treated with trastuzumab should improve our understanding of HER in breast pathogenesis and allow more accurate risk profiles and better treatment options to be developed.

## Competing interests

The authors declare that they have no competing interests.

## Authors' contributions

JAH performed all pathological characterizations, BD performed FISH analysis on all specimens, HP carried out the genotyping experiments, studies, BL oversaw all statistical analysis, DLE and CDS contributed to the preparation of the manuscript and REE conceived of the study, and participated in its design and coordination and helped to draft the manuscript. All authors read and approved the final manuscript.

## Pre-publication history

The pre-publication history for this paper can be accessed here:


